# Convergence of multimodal sensory pathways to the mushroom body calyx in *Drosophila melanogaster*

**DOI:** 10.1038/srep29481

**Published:** 2016-07-11

**Authors:** Ryosuke Yagi, Yuta Mabuchi, Makoto Mizunami, Nobuaki K. Tanaka

**Affiliations:** 1 Graduate School of Life Sciences, Hokkaido University, Sapporo, Japan; 2 Department of Biological Sciences, Hokkaido University, Sapporo, Japan; 3 Faculty of Science, Hokkaido University, Sapporo, Japan; 4 Creative Research Institution, Hokkaido University, Sapporo, Japan; 5 Precursory Research for Embryonic Science and Technology, Japan Science and Technology Agency, Saitama, Japan

## Abstract

Detailed structural analyses of the mushroom body which plays critical roles in olfactory learning and memory revealed that it is directly connected with multiple primary sensory centers in *Drosophila*. Connectivity patterns between the mushroom body and primary sensory centers suggest that each mushroom body lobe processes information on different combinations of multiple sensory modalities. This finding provides a novel focus of research by *Drosophila* genetics for perception of the external world by integrating multisensory signals.

The mushroom body (MB) is one of the prominent neuropils in the insect brain. Genetic manipulations of the MB intrinsic neurons called Kenyon cells in *Drosophila* have proved that the MB is an important site for olfactory learning and memory^1^,^2^,^3^. In contrast, the contribution of the *Drosophila* MB to general learning and memory is under debate. For example, flies in which postembryonic Kenyon cells were ablated by hydroxyurea showed normal visual, spatial, tactile, and motor learning, but were defective in olfactory learning^4^. Instead of the MB, the central complex is suggested to be the site of the memory trace of visual patterns^5^,^6^. Conversely, recent works based on the observation of flies in which neural transmission of Kenyon cells were blocked have shown that the MB is required for visual and gustatory learning^7^,^8^,^9^. To resolve these discrepancies, which probably reflect the different fly backgrounds and behavioral paradigms tested, accumulation of anatomical knowledge is indispensable. In *Drosophila*, it has been reported that only the olfactory antennal lobe (AL)^10^,^11^ and visual optic lobe (OL)^8^ are directly connected with the MB calyx where Kenyon cells receive sensory inputs. On the other hand, the MB calyx in other insects such as the honeybee is connected with not only the OL and AL, but also the gustatory subesophageal zone (SEZ)^12^,^13^. We thus analyzed the connectivity patterns between the primary sensory centers and MB calyx by dye injections and genetic labeling methods in *Drosophila*.

## Results

**Figure 1:**
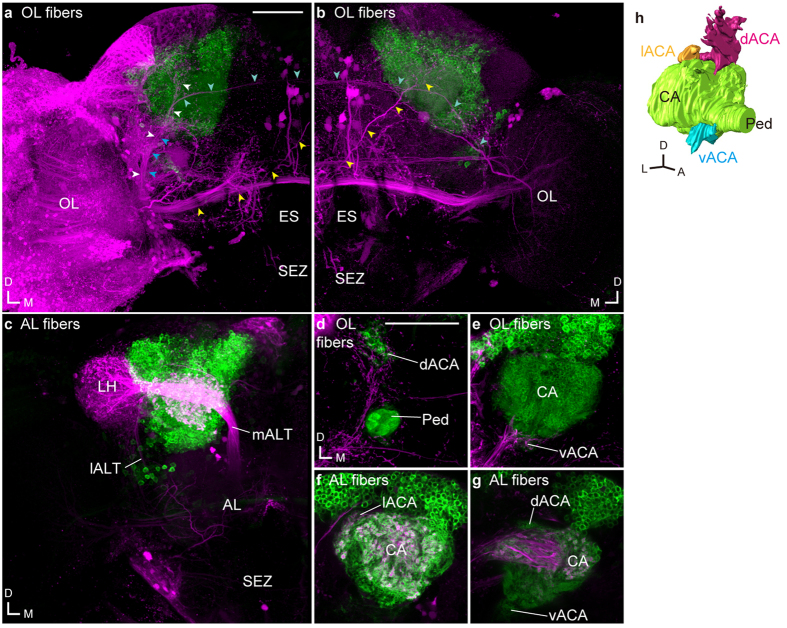
Sensory pathways visualized by dextran injection into the primary sensory center in *Drosophila*. Dextran conjugated with tetramethylrhodamine and biotin (magenta) was injected into the optic lobe (OL, (**a**,**b)**) or antennal lobe (AL, (**c)**) in flies expressing GFP (green) in Kenyon cells with OK107-GAL4. White, blue, and yellow arrowheads indicate the OLCT1, OLCT2, and OLCT5, respectively. Some OLCT2 neurons also innervate the contralateral hemisphere (cyan arrowheads). (**d–g**) The accessory calyces innervated by the labeled cells from the OL (**d**,**e**) and AL (**f**,**g**). (**h**) The oblique view of the mushroom body calyx labeled with OK107-GAL4. Green, magenta, yellow, and cyan represent the main calyx and pedunculus, dorsal, lateral, and ventral accessory calyx, respectively. The position of the lateral ACA was determined with the anti-SYNAPSIN signals. Scale bars = 50 μm. A, anterior; AL, antennal lobe; CA, main calyx; D, dorsal; dACA, dorsal accessory calyx; ES, esophagus; L, lateral; lACA, lateral accessory calyx; lALT, lateral antennal lobe tract; LH, lateral horn; M, medial; mALT, medial antennal lobe tract; OL, optic lobe; Ped, pedunculus; SEZ, subesophageal zone; vACA, ventral accessory calyx.

We performed injections of dextran conjugated with fluorescent dye into the primary sensory centers to investigate whether the MB calyx in *Drosophila* is also connected with multiple primary sensory centers. The MB calyx can be divided into four parts: the main calyx (CA), dorsal, lateral, and ventral accessory calyces (d-, l-, and vACA)^8^,^14^,^15^,^16^,^17^,^18^,^19^ (Fig. 1h and Supplementary Movie S1). The dye injections revealed that the MB calyx is connected with not only the AL and OL, but also SEZ via three major antennal lobe tracts (ALTs), three OL-calycal tracts (OLCTs), and one subesophageal-calycal tract (SCT) (Fig. 1a–g). The neurons running through the OLCTs are also called visual projection neuron (VPN)-MBs^8^. The terminals of ALTs were observed not only in the CA^10^,^11^, but also in the dACA and lACA. The OLCTs terminated in both dACA and vACA, whereas the SCT in dACA.

We then screened for the Janelia Farm GAL4 strains^14^ which visualize neurons running through these tracts and innervating the MB calyx except for the conventional ALT neurons which are known to supply branches into the main calyx^10^,^11^,^20^, subsequently identifying eleven strains, which labeled five OLCTs, two ALTs, and one SCT which send projections into the ACAs of the MB (Table 1). The dACA is innervated by two OLCTs, the medial ALT (mALT), and SCT. The lACA is innervated by one OLCT and the mediolateral ALT (mlALT) and the vACA by four OLCTs, respectively (Table 1 and Supplementary Fig. S1). Dye injection and labeling with the GAL4 lines showed that the terminal areas of OLCT and ALT neurons are overlapping in the lACA and posterior area of the dACA, however, are distributed to the vACA and CA, respectively, in a segregated manner (Fig. 1d–g).

**Figure 2:**
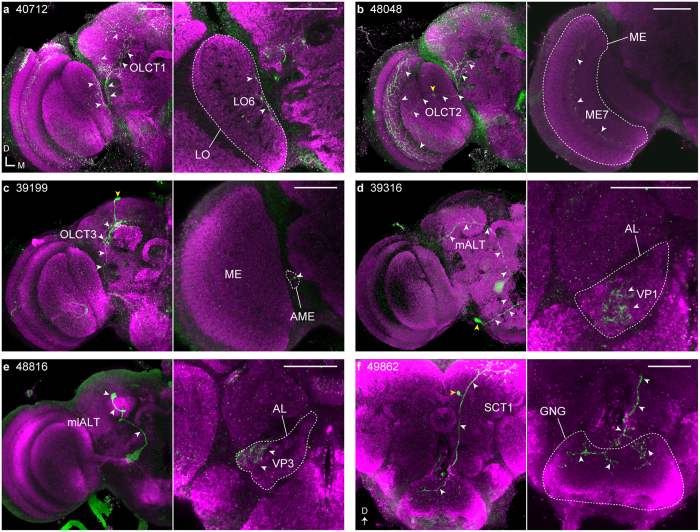
Single cell morphologies of the neurons which connect between the primary sensory centers and MB accessory calyx revealed by FLP-out experiments. Strain number of the Janelia Farm GAL4 line is shown top left in the left panel. Magenta shows the anti-SYNAPSIN antibody signals. Left panel: Single neuron (green) labeled with the GAL4 strain by the FLP-out experiment. The cell body and trajectory of the neural fiber from the primary sensory center to the calyx are indicated by yellow and white arrowheads, respectively. Right panel: Arborizations in the primary sensory center shown by arrowheads. (**a**,**b**,**d** and **e)** are single confocal images. AME, accessory medulla; GNG, gnathal ganglia; LO, lobula; ME, medulla; mlALT, mediolateral antennal lobe tract; OLCT, optic lobe calycal tract; SCT, subesophageal calycal tract. Genotype: *Hs-flp*;*UAS* > *CD2, y*^+^ >*CD8::GFP*;*GAL4.* Scale bars = 50 μm.

We further visualized the morphologies of single cells of these tracts by performing the FLP-out experiments^21^ using the GAL4 strains (Fig. 2, Supplementary Fig. S2, and Table 1). Most of the OLCTs emanated from the seventh layer of the medulla (ME7, serpentine layer), sixth layer of the lobula (LO6), or accessory medulla (AME). The ME7 and LO6 are the target layers of the chromatic Tm neurons^22^,^23^, suggesting that these layers are related to color processing. Only a few neurons innervate the layer entirely, however, most OLCT neurons have specific arborization areas in the OL. For example, one OLCT1 neuron innervates the dorsal three-quarters of the LO6 (Fig. 2a), whereas the innervation of one OLCT2 neuron is restricted to the ventralmost ME7 (Table 1). This result suggests that these neurons transfer information of specific visual fields. It should be noted that none of the OLCT neurons have the same morphologies with recently reported VPNs (VPN-MB1 and VPN-MB2) terminating in the vACA^8^. For the ALTs, we found two ALT neurons terminating in the ACA. One neuron, which innervates the VP1 glomerulus that receives chemosensory, hygrosensory, or thermosensory inputs from the sensory neurons in the sacculus of the antenna^19^,^24^, projects into the dACA through the mALT (Fig. 2d). The other, mlALT neuron, originating from the VP3 glomerulus which receives input from cold-sensing neurons^19^,^25^, runs through the mediolateral ALT and then pedunculus, and forms a cloud of terminals in the lACA, the structure of which is quite similar with the terminals of the tritocerebral tract in the orthopteran insects^26^ (Fig. 2e). The morphology of this mlALT neuron appears quite similar with that of the t5ALT neuron^14^,^19^, though the neuron we found runs through the mlALT. The SCT neuron originating from the gnathal ganglia (GNG) or flange (FLA) joins the mALT and sends branches into the dACA (Fig. 2f).

Taken together, the dACA is connected with the LO6, ME7, AME, VP1 glomerulus, FLA, and GNG. On the other hand, the vACA is linked with the LO6, ME1-7, and AME, whereas the lACA is linked with the AME and VP3 glomerulus. This indicates that each calycal part receives sensory inputs of different combinations of modalities and that the MB in *Drosophila* may play a role in general learning and memory.

**Figure 3:**
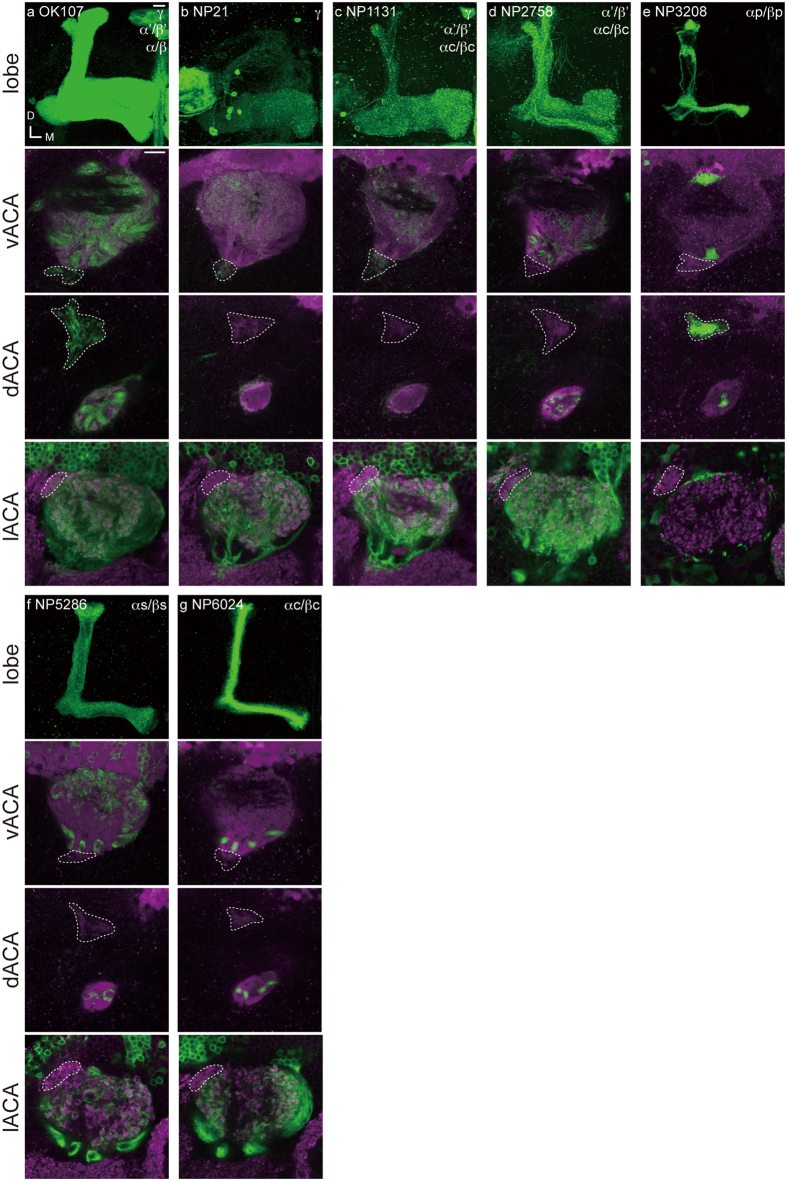
The connectivity patterns between the mushroom body accessory calyces and lobes by Kenyon cells. Top: The mushroom body lobes (green) labeled with the GAL4 enhancer-trap strain. Strain number and labeled Kenyon cell subpopulations are shown top left and top right, respectively. Bottom three panels: The mushroom body calyces innervated by Kenyon cell subpopulation shown in green. Magenta signals represent the entire calyx visualized with MB247-DsRed (vACA and dACA panels) and anti-SYNAPSIN antibody signals (lACA panels). The lACA is identified with the strong anti-SYNAPSIN antibody signals. The vACA (top), dACA (middle), and lACA (bottom) are indicated by dashed lines. Scale bars = 10 μm.

**Figure 4:**
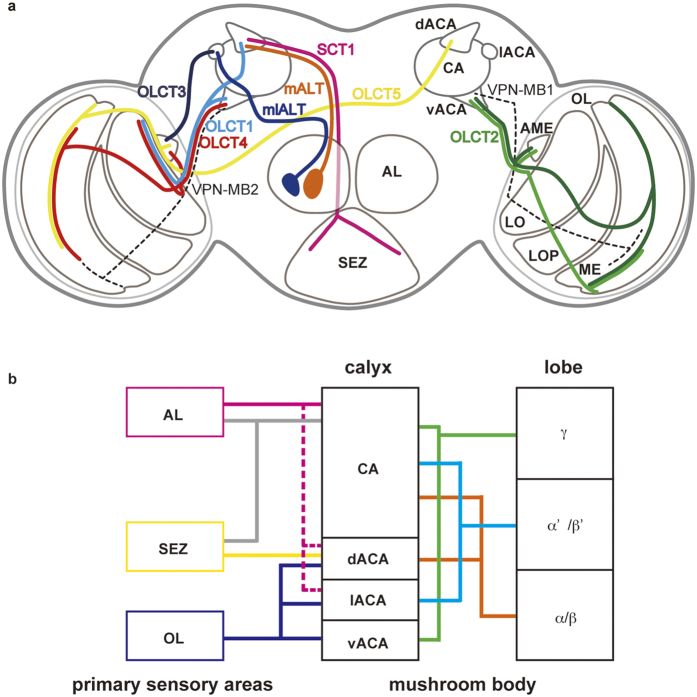
The connectivity pattern between the primary sensory centers and mushroom body. (**a**) Schema of sensory pathways to the MB ACA revealed by single cell analyses. It should be noted that branches outside of the primary sensory centers and MB calyx are not drawn in this schema. To reduce the complexity, the OLCT pathways as shown are divided betweeen the two hemispheres. The VPN-MB1 and VPN-MB2 which innervate the ME8 and ME7 respectively and terminate in the vACA^8^ are shown in dashed black lines. (**b**) Diagram of connectivity patterns of calycal parts with primary sensory centers and MB lobes. The antenno-subesophageal tract reported previously^11^ are shown in gray lines. Magenta dashed lines represent the thermosensory pathways^19^. LOP, lobula plate.

We furthermore investigated how each calycal part is governed by different classes of Kenyon cells. Kenyon cells are classified according to the lobes they terminate in: γ, α/β, or α’/β’ lobe neurons^15^,^16^,^17^,^27^,^28^. We analyzed the dendritic patterns of these neurons by using the GAL4 enhancer-trap strains we previously reported^28^. Since there were no available strains for labeling α’/β’ lobe neurons specifically, we compared staining patterns among the strains labeling Kenyon cells of multiple lobes to estimate the probable innervation areas of α’/β’ lobe neurons. Each population of γ, α/β, or α’/β’ lobe neurons arborizes in the CA, whereas the ACAs are innervated by specific populations (Fig. 3 and Table 2). The dACA is innervated by a subpopulation of α/β lobe (αp/βp) Kenyon cells^28^, whereas the vACA is innervated by γ lobe neurons^8^,^15^,^16^,^17^. However, the innervation into the lACA was not observed in those strains that specifically label γ or α/β lobe neurons, but strains which visualize α’/β’ lobe neurons, suggesting that the lACA is innervated by α’/β’ lobe neurons. Since each ACA is connected with different primary sensory centers, these anatomical findings suggest that each lobe may have different functions in multimodal sensory processing (Fig. 4).

## Discussion

This study reveals multimodal sensory pathways into the MB calyx in *Drosophila* as has been reported in such insects as the honeybee, ant, butterfly and cockroach^12^,^13^,^29^,^30^. Recent work has also revealed that two other OLCT neurons, VPN-MB1 and MB2 are required for visual memories of color and brightness in *Drosophila*^8^. In the bumblebee and honeybee, the neurons of the anterior inferior optic tract and the lobula tract which arborize in the serpentine layer of the medulla and in LO5 or LO6 respectively and project to the MB calyx respond to color and motion stimuli^31^. Due to morphological similarities of the OLCT neurons found in this study to these neurons and VPN-MBs^8^, the *Drosophila* MB may also receive similar visual information and combine it with odor, gustatory, and temperature information in each lobe. Recent physiological works support this idea: the γ lobe neurons innervating the vACA respond to visual stimuli^8^, the ALT neuron terminating in the lACA shows calcium responses to temperature shifts^19^, and taste activity has been observed in the dACA^9^. Further physiological works revealing the sensory information transferred from the primary sensory centers to the MB calyx by the neurons found in this study need as yet to be determined.

Previous works have shown that each MB lobe contributes to a different temporal phase of memory^1^,^2^. This study suggests that each lobe functions in integrating a different combination of multiple sensory modalities. In the cockroach, the MB extrinsic neurons which innervate the MB lobe respond to multimodal sensory inputs in a context-specific manner^32^. Readily available *Drosophila* genetics^17^,^18^,^28^ can provide opportunities to reveal novel neural mechanisms by which a rich variety of sensory cues are combined to represent coherent perception of the external world.

## Methods

### *Drosophila* strains

To visualize the MB, OK107^33^, six GAL4 enhancer-trap strains^28^ (obtained from Kyoto *Drosophila* Genetic Resource Center), and MB247-DsRed line^34^ (gifted from Thomas Riemensperger and Andre Fiala) were used. To label the neurons which connect the primary sensory center and the MB calyx, we screened for about 6000 Janelia Farm GAL4 strains^14^,^35^ from the expression data of Bloomington *Drosophila* stock center (BDSC) website (http://flystocks.bio.indiana.edu/Browse/gal4/gal4_Janelia.php) and identified eleven strains. These strains were crossed with a strain carrying *UAS-GFP*, *UAS-mCD8::GFP*^36^ gifted from Aki Ejima, or *20xUAS-IVS-mCD8::GFP*^37^ (#32194, BDSC). To reveal the morphologies of single neurons (see below), the Janelia Farm GAL4 strains were crossed with flies carrying *Hs-flp* and *UAS* > *CD2, y*^+^ >*CD8::GFP*^21^ kindly provided by Gary Struhl. Except for the FLP-out experiments, flies were raised at 25 degrees Celsius with a 12-hour light/12-hour dark cycle. We analyzed female flies between 2 and 8 days after eclosion.

### Dextran injection into the primary sensory centers

Flies were anesthetized in a vial on ice for less than a minute and were fixed to plastic chambers with wax and epoxy. The compound eye, top of the head, or proboscis was opened with forceps in *Drosophila* saline (in mM: NaCl 103, KCl 3, MgCl_2_ 4, CaCl_2_ 1.5, NaHCO_3_ 26, TES 5, trehalose 10, glucose 10, sucrose 7, NaH_2_PO_4_ 1, adjusted to pH 7.25 with HCl). After fat, air sacs, and sheathes around the OL, AL, or SEZ were gently removed with forceps, dextran conjugated with tetramethylrhodamine and biotin (3 kDa, D-3308, Life Technologies) was injected into one of the OL, AL and SEZ with forceps^20^. Within 20 minutes after dextran injection, the brains were dissected out and fixed with 4% formaldehyde or 4% paraformaldehyde (PFA) for 50 minutes at room temperature. The brains were then washed in phosphate buffered saline (PBS, pH 7.4). When the GFP and tetramethylrhodamine signals needed to be enhanced, the brains were further incubated in the rabbit anti-GFP antibody and Alexa Fluor 568-conjugated streptavidin (S-11226, Life Technologies; 1 mg/l diluted in 0.2% Triton X-100/PBS (PBST)), respectively, followed by the antibody staining procedures (see below). The brains were finally mounted in 50 or 80% glycerol/PBS.

### Antibody staining

Brains were dissected out in PBS and fixed with 4% PFA for 50 minutes. After washing with PBS for 5 minutes three times, the brains were then shaken in the blocking solution: 10% goat serum in PBST for an hour. They were incubated in the blocking solution containing primary antibodies overnight at room temperature with shaking. After washing with PBST, the brains were then incubated in the blocking solution containing secondary antibodies overnight at room temperature with shaking. Finally, the brains were washed with PBS and mounted in 50 or 80% glycerol/PBS.

For primary antibodies, we used rabbit anti-GFP antibody (A11122, Life Technologies; diluted at 1:200 except for the FLP-out experiments, see below), mouse anti-GFP antibody (12A6, Developmental Studies Hybridoma Bank at the University of Iowa; diluted at 1:200), or mouse anti-SYNAPSIN antibody^38^ (3C11, Developmental Studies Hybridoma Bank at the University of Iowa; diluted at 1:10). For secondary antibodies, we used goat anti-rabbit/mouse antibodies conjugated with Alexa Fluor 488 or 568 (A11001, A11004, A11034, and A11036, Life Technologies; diluted at 1:200), except for the FLP-out experiments (see below).

### Labeling single neurons with GFP

Janelia Farm GAL4 strains labeled multiple neurons including our targets, which prevented us from analyzing the morphology of single cells. To reduce the number of neurons labeled, we used the FLP-out system^21^. In this system, we crossed each GAL4 strain with the flies carrying *Hs-flp* and *UAS* > *CD2, y*^+^ >*CD8::GFP* and incubated one-day old adult offspring at 37 degrees Celcius for 1 hour to express the *flippase* gene to induce somatic recombination randomly, which resulted in GFP expression in a fewer numbers of neurons. We then maintained the flies for 6–8 days at 25 degrees Celcius before dissecting the brains out. The brains were then fixed with 4% PFA, and immunostained with the rabbit anti-GFP antibody (diluted at 1:1000) and the mouse anti-SYNAPSIN antibody (diluted at 1:20). For secondary antibodies, we used goat anti-rabbit antibody conjugated with Alexa Flour 488 (diluted at 1:1000) and goat anti-mouse antibody conjugated with Alexa Flour 568 (diluted at 1:200). The brains were washed with PBS and finally mounted in 80% glycerol/PBS. The brains expressing CD8::GFP in a single neuron of interest were imaged.

### Confocal imaging and three-dimensional reconstructions of optical sections

Confocal serial optical images of the whole-mount brain were taken at 0.7–1 μm z-intervals with a Carl Zeiss confocal microscope LSM 700 equipped with water-immersion C-Apochromat 40×. Three-dimensional reconstruction and surface rendering were performed with Zeiss ZEN 2012 and Avizo 5.1, respectively. The brightness, color, and contrast of images were adjusted with Photoshop CS 5.1. For the left panels of Fig. 2d–f, the antibody signal levels of the fibers we analyzed were adjusted to increase the signal/noise ratio by changing the levels manually in Photoshop before the reconstructions. For the right panels of Fig. 2f, antibody signals except for the SCT1, such as the noise backgrounds on the surface of the brain were manually removed to visualize the GAL4 positive neuron more clearly.

### Terminology

The spatial definition and nomenclature of each lobe and axonal tract is based on ref. 39. The layer divisions in the medulla and lobula are referred to ref. 40.

## Additional Information

**How to cite this article**: Yagi, R. *et al*. Convergence of multimodal sensory pathways to the mushroom body calyx in *Drosophila melanogaster.* *Sci. Rep.* **6**, 29481; doi: 10.1038/srep29481 (2016).

## Supplementary Material

Supplementary Information

Supplementary Movie S1

## Figures and Tables

**Table 1:** Neurons that connect primary sensory centers and the mushroom body accessory calyx revealed by single cell analyses.

Cell type	Strain number	mushroom body calyx	primary sensory center	projections outside MB calyx	cell body
OLCT1	40712	dACA#, vACA	OL(LO6*)	ICL, PLP, SLP, SCL	near AME
OLCT2	48048	vACA	OL(ME7)	PLP	near AME
OLCT2	38695	vACA	OL(AME, ME7**)	PLP, SPS	near AME
OLCT2	38866	vACA	OL(AME, ME7)	PLP	near AME
OLCT2	49421	vACA	OL(AME,ME1-5**, ME6-7***)	PLP, ICL	near AME
OLCT3	39199	lACA	OL(AME)	PLP, SLP	SLP
OLCT4	40712	vACA	OL(AME, LO6, ME7)	ICL, PLP, WED	near AME
OLCT5	40596	dACA, vACA	OL(AME, ME7*)	ATL, ICL, PLP, SCL, SLP, SMP	SPS
mALT	39316	dACA##, CA	AL(VP1)	LH	GNG
mlALT	48816	lACA	AL(VP3)		GNG
SCT1	49862	dACA##	GNG	SLP, SMP, LH	posterior to PB
SCT1	49910	dACA##	FLA	SLP, SMP, LH	posterior to PB

Numbers following the ME and LO represent the layer numbers in which the neurons arborize. # and ## show that the neuron arborizes only in the anterior (#) and posterior (##) part of the dACA, respectively. Asterisks represent that the neurons which innervate only in the dorsal three-quarters (*), ventralmost (**), or ventral half (***) of the lobula or medulla were observed. AL, antennal lobe; AME, accessory medulla; ATL, antler; CA, main calyx; AVLP, anterior ventrolateral protocerebrum; dACA, dorsal accessory calyx; FLA, flange; GNG, gnathal ganglia; ICL, inferior clamp; lACA, lateral accessory calyx; LH, lateral horn; LO, lobula; mALT, medial antennal lobe tract; ME, medulla; mlALT, mediolateral antennal lobe tract; OL, optic lobe; OLCT, optic lobe calycal tract; PB, protocerebral bridge; PLP, posterior lateral protocerebrum; PRW, prow; SCL, superior clamp; SCT, subesophageal calycal tract; SLP, superior lateral protocerebrum; SMP, superior medial protocerebrum; SPS, superior posterior slope; vACA, ventral accessory calyx; WED, wedge.

**Table 2:** GAL4 enhancer-trap strains that label Kenyon cells.

strain number	γ	α′/β′	α/β	CA	dACA	lACA	vACA
OK107	entire	entire	entire	+	+	+	+
NP21	entire	−	−	+	−	−	+
NP1131	entire	entire	core	+	−	+	+
NP2758	−	entire	core	+	−	+	−
NP3208	−	−	posterior	−	+	−	−
NP5286	−	−	surface	+	−	−	−
NP6024	−	−	core	+	−	−	−

It should be noted that more lobes were labeled with NP1131 and NP2758 than those in our previous work^28^, possibly because the GFP expression is enhanced by the repeats of UAS in this work.
