# Supplementary material for: Convergence of multimodal sensory pathways to the mushroom body calyx in Drosophila melanogaster
Source: Sci Rep. 2016 Jul 11;6:29481. doi: 10.1038/srep29481 (PMC4941532; doi:10.1038/srep29481)
Supplement: Supplementary Information [file srep29481-s1.pdf]

Convergence of multimodal sensory pathways to the mushroom body calyx in *Drosophila melanogaster*

Ryosuke Yagi, Yuta Mabuchi, Makoto Mizunami, and Nobuaki K. Tanaka

Supplementary Movie S1. The mushroom body calyx of the right hemisphere labeled with *OK107-GAL4*. Green, magenta, yellow, and cyan represent the main calyx and pedunculus, dorsal, lateral, and ventral accessory calyx, respectively. It should be noted that there are two more protrusions from the main calyx, which may also be the accessory calyces receiving other modalities of sensory information than olfaction.

Supplementary Fig. S1. Janelia Farm GAL4 strains which visualize the sensory pathways to the mushroom body accessory calyces. Strain number is shown top left in the left panel. **Left panel:** GAL4 expression pattern (GFP) in the left hemisphere. The cell bodies and axonal pathways are indicated by yellow (**f**) and white arrowheads (all panels), respectively. Since many neurons other than the sensory pathway neurons obscure the sensory pathways, the full trajectories are not indicated by arrowheads.

Magenta represents the mushroom body. **Right panel:** Innervation into the calycal region (arrowheads). Genotype: *MB247-DsRed*; *GAL4*/*20xUAS-mCD8::GFP*. Scale bars = 50 µm. CA, main calyx; D, dorsal; dACA, dorsal accessory calyx; lACA, lateral accessory calyx; M, medial; mALT, medial antennal lobe tract; mlALT, mediolateral antennal lobe tract; OL, optic lobe; OLCT, optic lobe calycal tract; Ped, pedunculus; SCT, subesophageal calycal tract; vACA, ventral accessory calyx.

Supplementary Fig. S2. The OLCT4 and 5 neurons which connect the OL and MB calyx. Single cells of each type are shown in 3D reconstruction. The axonal pathway and cell body are indicated by white and yellow arrowheads, respectively. (**a**) The OLCT4 neuron in the left hemisphere (left) and in the optic lobe (right). (**b**) The OLCT5 neuron innervates both hemispheres and terminates in the wide protocerebral area. Left and right panels are taken from the same preparation. Scale bars = 50 µm.

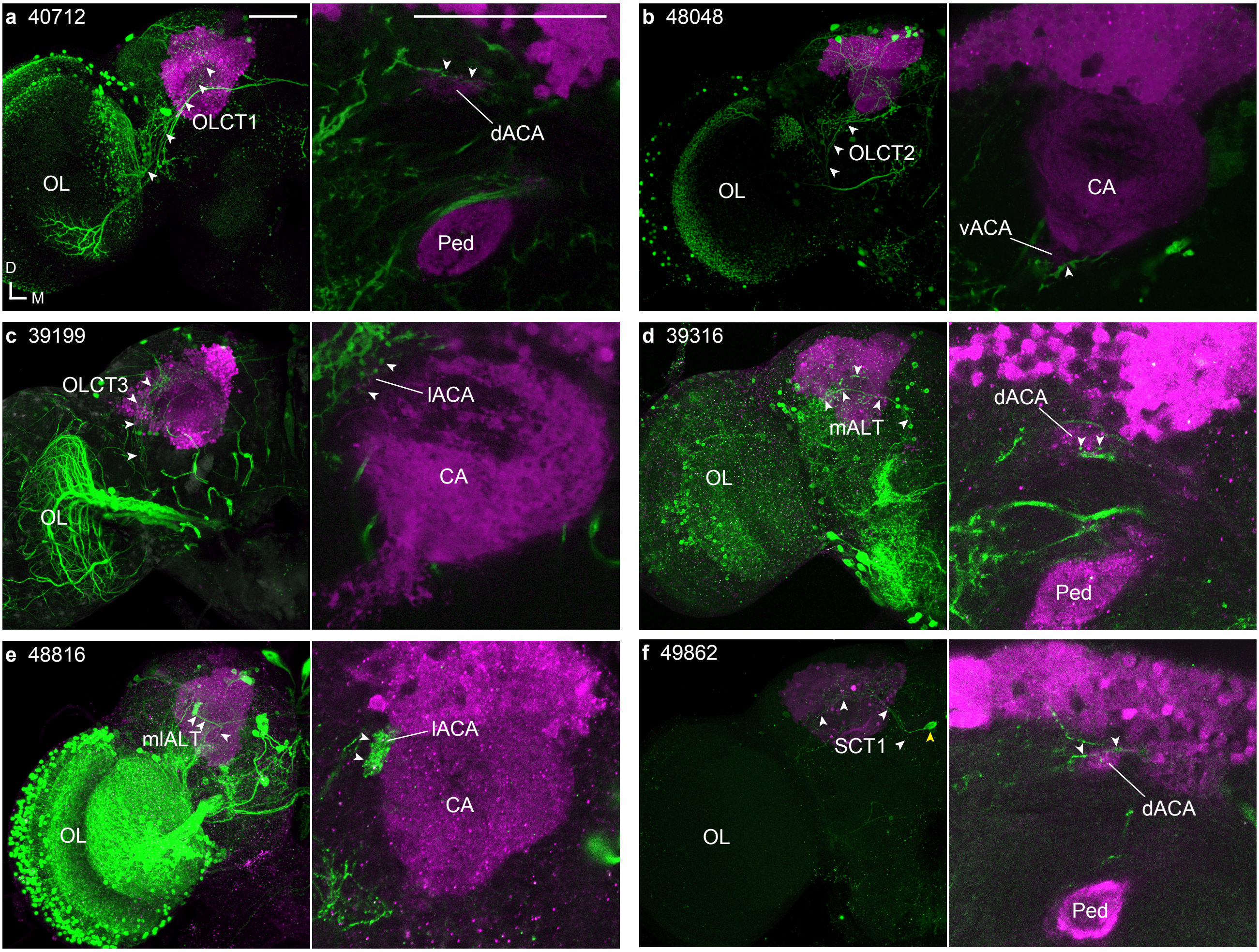

Supplementary Fig. S1

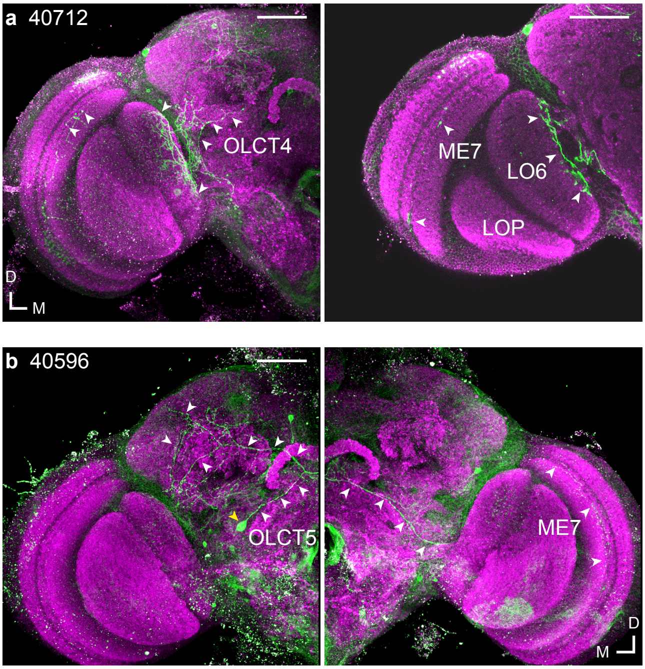

Supplementary Fig. S2
